# Epididymal-Born circRNA Cargo and Its Implications in Male Fertility

**DOI:** 10.3390/ijms26062614

**Published:** 2025-03-14

**Authors:** Francesco Manfrevola, Nicola Mosca, Vincenza Grazia Mele, Teresa Chioccarelli, Antonella Migliaccio, Monica Mattia, Mariaceleste Pezzullo, Gilda Cobellis, Nicoletta Potenza, Rosanna Chianese

**Affiliations:** 1 Department of Experimental Medicine, University of Campania “Luigi Vanvitelli”, 80138 Naples, Italy; francesco.manfrevola@unicampania.it (F.M.); vincenzagrazia.mele@unicampania.it (V.G.M.); teresa.chioccarelli@unicampania.it (T.C.); antonella.migliaccio@unicampania.it (A.M.); monica.mattia@unicampania.it (M.M.); gilda.cobellis@unicampania.it (G.C.); 2 Department of Environmental, Biological and Pharmaceutical Science and Technologies, University of Campania “Luigi Vanvitelli”, 81100 Caserta, Italy; nicola.mosca@unicampania.it (N.M.); mariaceleste.pezzullo@unicampania.it (M.P.); nicoletta.potenza@unicampania.it (N.P.)

**Keywords:** epididymis, circRNAs, backsplicing, male fertility, aging

## Abstract

The epididymis represents a pivotal organ for sperm maturation and male fertility maintenance. During the epididymal journey, sperm cells undergo morphological and molecular changes that need to acquire the morpho-functional skills necessary for successful oocyte fertilization. Not last, a great enrichment of the spermatozoa RNA payload occurs via an epithelium-derived epididymosome transfer. Currently, circular RNAs (circRNAs), a class of non-coding RNAs (ncRNAs), are acquiring a prominent role in the setting of sperm quality parameters. In this regard, they are considered potential targets in several male infertility conditions. Despite their consolidated role, few notions are known regarding the alleged epididymal backsplicing activity. In the current review, we discuss the main aspects of spermatozoa maturation along the epididymis and the circRNA role in the field of male reproduction. We also report the most recent findings on the circRNA biogenesis that occurs in the epididymal duct, providing new fascinating evidence on epididymal-derived circRNAs. Finally, we show preliminary compelling data on epididymal backsplicing by exploiting the experimental mouse model of aging. Collectively, these data evidence a remarkable role of the epididymis in remodeling the circRNA payload and in shaping its profile in maturating spermatozoa.

## 1. Introduction

Spermatogenesis is a highly complex biological process that occurs in the testis, which is responsible for spermatozoa production. Beyond the testis, immature spermatozoa transit along the epididymal duct undergoing the deep molecular and morpho-functional changes needed to acquire fertilizing abilities [1,2]. Functionally, during the epididymal journey, spermatozoa acquire motility, whereas at the morphological level, they modify the distribution of surface proteins involved in sperm–oocyte binding and expel residual cytoplasmic droplets. More importantly, the continuous exchange of molecules within the epididymal lumen, such as ions and antioxidants, promotes sperm nourishing and protection [1,3,4]. In addition, the spermatic uptake of epididymosomes, which consist of epididymal-derived exosomes released by the epididymal epithelium, leads to a substantial enrichment in the sperm transcriptome and proteome [4,5,6,7,8,9,10]. As a consequence, any inadequate epididymal mechanism and physio-pathological condition attributable to it can compromise sperm maturation, leading to several dysfunctions. In this context, circular RNAs (circRNAs) are gaining growing scientific interest. Indeed, recent findings highlighted the modulation of spermatic circRNAs depending on the (i) morpho-functional quality parameters, such as sperm structural integrity and motility [11]; (ii) asthenozoospermia and obesity pathological conditions [12,13,14,15]; (iii) sperm actin remodeling dynamics [16]; and (iv) teratozoospermic phenotype related to environmental contaminant exposure [17].

Despite this background, a functional link between circRNAs and sperm epididymal maturation is under-investigated. In this review, we summarize the main aspects regarding sperm epididymal maturation, focusing on the essential role of the epididymis in ensuring sperm reproductive skills. Additionally, we outline the role of circRNAs in the field of male reproduction, showing new fascinating insights into epididymal-derived circRNAs. In detail, we report the most recent findings on circRNA biogenesis that occurs in the epididymal duct obtained from the study of poor fertility animal models, including data that resulted from a mouse model of aging, shown here for the first time.

## 2. Epididymal Structure

**Figure 1:**
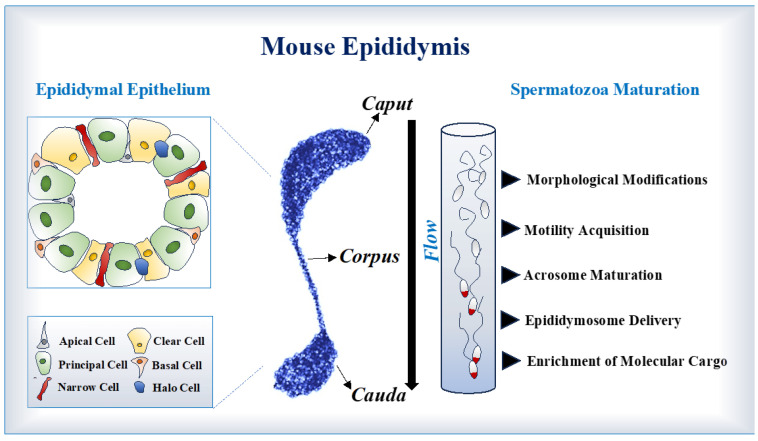
An overall view of the mouse epididymis. Representation of the anatomical regions (*caput*, *corpus* and *cauda*) of the mouse epididymis. Epididymal epithelium cell types and sperm maturation occur along the epididymis, as schematized on the left and on the right sides, respectively.

Sperm maturation occurs along the epididymis [10]. This organ consists of an elongated and convoluted duct, connecting efferent ducts to the vas deferens, mainly divided into four anatomical regions: the initial segment, *caput*, *corpus* and *cauda* (Figure 1) [18,19,20,21]. Morphologically, the epididymal epithelium contains several cell types whose metabolic, secretory and endocytic activities ensure the functionality of the organ [4,19,22]. The major cell type in the epididymal duct is represented by Principal cells, which appear along the entire epithelium length with different density grade depending on the epididymal anatomical region. These cells especially modulate the secretion of epididymosomes, extracellular vesicles containing a cargo of proteins and RNAs to be carried by the spermatozoa in transit [4,6,7,19]. The Narrow cells regulate the secretion of H^+^ ions into the epididymal lumen involved in the modulation of the endocytic activity. Similarly, the Apical cells, located at the initial segment of the epididymal epithelium, regulate the transport of electrolytes and, in turn, the epididymal fluid pH [22,23]. The Basal cells are primarily located in the initial and intermediate regions, adhering to the basement membrane, and participate in the luminal environment control [24,25,26,27,28,29]. Finally, the Clear cells, present in the *caput*, *corpus* and *cauda* regions, take part in the endocytic mechanism modulating the absorption of proteins from the lumen, whereas the Halo cells represent the immunological cellular counterpart of the epididymis [22,27,30].

The intricate architecture of the epididymal epithelium is ensured by the establishment of the epithelial intercellular junctions (tight, adherens and gap junctions) that underlie the functionality of the Blood–Epididymal Barrier (BEB) [7,10,31,32]. The BEB acts as an epididymal immunological barrier that ensures the appropriate microenvironment needed to prevent harmful autoimmune responses. In addition, the BEB—by regulating epididymal lumen acidification—promotes the morpho-functional sperm maturation along the epididymal duct and, not least, the proper biogenesis and transfer of the epididymosomes from the epithelial cells to spermatozoa [7,10,31]. As a consequence, any BEB structural alterations may lead to male infertility conditions.

In this context, the main epithelial intercellular junctions participating in the BEB integrity consist of (i) the tight junctions (TJs), which are the most prevalent intercellular junctions established among adjacent Principal cells via the interaction of several transmembrane proteins, such as claudins (CLDNs), intracellular zona occludens proteins (ZOs) and occludins (OCLNs) [32,33]; (ii) the adherens junctions, which are formed by cadherin and nectin proteins [32,34,35]; and (iii) the gap junctions, which mainly occur between Principal cells and Basal cells, as well as between Clear cells and Basal cells, and they are molecularly dependent on connexin proteins [32,36].

## 3. Sperm Epididymal Maturation

The principal function of the epididymis consists of the transport of spermatozoa from the rete testis to the vas deferens [27]. Sperm epididymal transit is achieved by the contractions of smooth muscle layers surrounding the epididymis, which also modulate the direction of transit [27,37]. During the epididymal maturation, spermatozoa undergo morphological and molecular changes to acquire the progressive motility and molecular factors necessary for a successful oocyte fertilization. All processes that occur during the epididymal transit are finely regulated by the epididymal lumen environment, which depend on the epididymal region and finely orchestrate each spermatic feature [19,27]. The acquisition of sperm motility is associated with a vast set of morphological modifications. Indeed, the modulation of the sperm plasma membrane lipidic composition; the migration of cytoplasmic droplet along sperm tail; and finally, the activation of cytoskeletal flagellar machinery represents the main morphological changes that lead to proper motility acquisition [3,19]. In addition to the latter, spermatozoa also display molecular factors that mediate zona pellucida penetration and the binding to the oocyte. This fundamental molecular interaction underling sperm–oocyte binding occurs between receptor proteins localized on the sperm membrane and oligosaccharides expressed on the oocyte membrane [38,39]. In this context, many proteins localized on the acrosome surface are actively involved in sperm–oocyte binding, and most of them molecularly maturate during the epididymal transit. Among them, worthy of note are sperm–zona binding (ZP) proteins, ADAM family proteins, acrosin, IZUMO1 and Binder of SPerm (BSP) family proteins [38,40,41,42,43].

Additionally, along the epididymis, spermatozoa are protected from the reactive oxygen species (ROS)-mediated damage derived from the external environment. Indeed, many antioxidant enzymes are produced by the epididymal epithelial cells and released into the epididymal lumen in order to neutralize the ROS [44]. Accordingly, the BEB protects sperm from harmful immune responses [7,10,27,31,32,45].

Lastly, the epididymis acts as a storage site for mature spermatozoa before ejaculation. Interestingly, during caudal storage, spermatozoa persist in a quiescent state that is ensured by the secretion of specific epididymal-derived factors into the lumen [19,27]. In this scenario, the epididymal secretory activity also modulates the enrichment of the spermatic protein and non-coding RNA (ncRNA) payload [42].

## 4. CircRNAs and Male Fertility

CircRNAs consist of covalently closed single-stranded circular molecules derived from a backsplicing mechanism. This latter, which promotes the inter-site covalent closure between the downstream (3′) splice donor site and the relative upstream (5′) splice acceptor site, is finely orchestrated by several RNA-binding proteins (RBPs), especially by the fused in sarcoma (FUS) and quaking (QKI) proteins [46,47,48]. The circRNAs were identified in (i) mammalian testis, (ii) seminal plasma and (iii) spermatozoa, thus evidencing a key role for these molecules in the regulation of male fertility (Table 1) [11,12,13,14,16,49,50,51,52,53,54,55,56,57,58,59].

In support of this, by using sophisticated high-throughput sequencing techniques, selective circRNA expression patterns were profiled in several fertility experimental models. Zhou and co-authors have reported the expression of circRNAs in a testicular spermatogonial stem cell population, highlighting the involvement of these molecules in the control of the male germ cell lineage [58,60]. Accordingly, the importance of circRNAs in spermatogenesis and testis development was also shown in a goat animal model [61]. In Holstein bull’s testes, 3.032 differentially expressed (DE)-circRNAs bioinformatically predicted to regulate chromosome segregation, sperm tail formation and sperm motility were identified [62], whereas a set of 2.326 DE-circRNAs potentially implicated in spermatogenesis regulation and sperm motility were profiled in boar testis [63]. Accordingly, the global transcriptomic analysis performed in goat spermatozoa has highlighted spermatogenesis-related circRNAs [64].

In addition, the detection of testis-derived circRNAs in human seminal plasma suggests their potential applicability as biomarkers for various fertility diseases. Interestingly, they are bound to proteins and carried through exosomes [49,54,55]. However, alternative transfer mechanistic pathways of testis-derived circRNAs, such as cell-to-cell contact, are poorly investigated.

To date, scientific evidence has highlighted a new emerging role of circRNAs as spermatic markers of quality. In human spermatozoa, within a cargo of 10.726 circRNAs, a set of 148 appeared differentially expressed relative to the cellular morphology and motility grade [11]. In the context of sperm quality parameters, El-Gamal and colleagues have reported circANKLE2 and circL3MBTL4, which were up- and downregulated in immature spermatozoa collected from normozoospermic men, respectively [65]. In agreement, the potential role of testis-specific circBOULE as a biomarker for sperm quality has been recently reported. Then, the expression of circEx2-6 negatively correlates with the sperm DNA fragmentation index, thus acting as a prognostic marker of sperm DNA quality [66].

In addition, in spermatozoa, preferential subcellular localization in the head or tail was demonstrated, suggesting a putative paternal-derived circRNA delivery to the embryo [11,67]. This hypothesis was confirmed by the profiling of circRNAs in both murine and human preimplantation embryos. Indeed, the dynamic modulation of their expression during embryo developmental stages (mainly in the four-to-eight-cell transition) and their potential involvement in the regulation of chromosome organization, cell cycle regulation and DNA repair mechanisms support this hypothesis well [68,69,70]. The first evidence of this came from the studies of Ragusa and colleagues that showed for the first time the contribution of paternal circRNAs to the zygote. Indeed, they revealed the physical interaction between the sperm-derived circNAPEPLDiso1 and miRNAs (miR-146a-5p, miR-203a-3p, miR-302c-3p, miR-766-3p and miR-1260a) primarily implicated in the control of the cell cycle and in the modulation of initial embryo development stages [67].

Subsequent studies in murine and human spermatozoa further confirmed the alleged contribution of paternal-derived circRNAs to embryo development. Indeed, it was experimentally proved that circCNOT6L, expressed in spermatozoa and not in oocytes, exerts a regulatory role in the zygote transition toward the two-cell-like state in the Embryonic Stem Cell (ESC) system [71]. Furthermore, both murine and human spermatozoa possess an intrinsic endogenous ability to produce circRNAs that are molecularly modulated by the interactive FUS-QKI-RNApol2 protein complexome [71]. Therefore, it is highly conceivable that any interference in the spermatic circRNA cargo can compromise paternal transgenerational epigenetic inheritance, impairing offspring health downstream.

In this scenario, several physio-pathological conditions that affect spermatic circRNA cargo have been reported (Table 2). Asthenozoospermia, which consists of the reduction in total (40%) and progressive (32%) sperm motility, promotes 1.432 spermatic DE-circRNAs, which are mainly involved in circRNA/miRNA/mRNA (ceRNET) networks that control sperm motility, as demonstrated by their responsiveness following amino acid supplementation therapy, which is commonly used to improve sperm motility [12,13]. In agreement, a set of circRNAs (circTRIM2, circEPS15, circRERE) was found to regulate the master genes (CRISP2, CATSPER1, PATE1) of sperm motility [72,73,74,75,76]. Similarly, the profiling of circRNAs in testicular tissue from Yili geese animal models with high and low sperm motility showed circRNAs associated with variations in sperm motility [77]. Interestingly, the impairment of the circANKLE2 and circL3MBTL4 expression profiles occurs in asthenozoospermia, astheno-teratozoospermia and oligo-astheno-teratozoospermia, whereas reduced levels of selective circBOULE were observed in asthenozoospermic (circEx3-6) and teratozoospermic (circEx2-6 and circEx2-7) patients [65,66].

Regarding non-obstructive azoospermia (NOA), a pathological condition in which testes are unable to produce spermatozoa, high-throughput circRNA microarray analyses performed in the testes of NOA patients compared with normal controls led to the identification of a vast set of DE-circRNAs implicated in axoneme assembly and microtubule-based processes [55,78,79]. In addition, the selective expression of circRNAs in whole blood and seminal plasma of idiopathic NOA patients has also been reported [75,80]. In this context, DE-circRNAs also play a key role in Sertoli cell-only syndrome (SCOS), a subtype of NOA characterized by the absence of germ cells in the seminiferous tubules. Indeed, Zhu and co-authors identified 1.594 DE-circRNAs expressed in SCOS testes compared with NOA patients [52] that were potentially implicated in Sertoli cell and spermatogenic microenvironment dysfunctions.

Obesity represents another pathological condition that affects male fertility. Studies carried out on male mice fed with a high-fat diet (HFD) showed 109 DE-circRNAs in spermatozoa collected from HFD mice compared with healthy controls [14]. The affected spermatic circRNA cargo is dependent on two distinct pathways: (i) increased sperm backsplicing activity and (ii) inefficient epididymal-derived circRNA release. In addition, in vivo studies stressed the involvement of circRNAs in the triggering of spermatic oxidative stress pathways observed in the HFD condition, confirming a direct role of these molecules in modulating the sperm quality parameters [15].

It is noteworthy that circRNAs have been reported to be ideal candidates useful for assessing decreased fertility skills dependent on the advanced paternal age (APA). RNA sequencing analyses performed in spermatozoa collected from APA vs. younger men identified 1.056 down- and 1.228 upregulated circRNAs, which were mainly involved in DNA repair and meiotic recombination pathways, thus suggesting a new intriguing role for these molecules in the regulatory mechanisms underlying the sperm quality in aged men [81].

## 5. CircRNAs and Epididymis: New Insights into Epididymal Backsplicing

The drawing of the spermatozoa RNA landscape is an intriguing multi-faceted topic; in this regard, the epididymis plays a key role. The epididymal epithelial cells, and in particular, the Principal cells, are responsible for the production and secretion into the lumen of epididymosomes. Such epididymal-derived exosomal vesicles physically interact with transiting spermatozoa to dissolve and release them into the containing cargo [6,82]. In terms of the RNA content, the epididymosomes appear particularly enriched with ncRNAs, including (i) microRNAs (miRNAs), (ii) piwiRNAs (piRNAs) and (iii) tRNA- and rRNA-derived fragments (tRFs and rRFs, respectively) [5,83,84,85]. Interestingly, the abundance of the spermatozoa ncRNA cargo undergoes a significant modulation along the epididymis, and the epididymosomes actively mediate these changes, which are necessary to ensure multiple downstream functions [4].

Several studies demonstrated that the enrichment of selected miRNAs and tRFs that occurs in caudal spermatozoa ensures embryo development [84,86,87,88,89,90,91]. As a consequence, pathological conditions, as well as harmful lifestyles and environmental factors, including diet and stress, can induce a profound variation of the spermatozoa ncRNA cargo. The impairment of epididymosome-dependent miRNA and tRF delivery compromises offspring health. Indeed, an unbalanced paternal diet increases the incidence of metabolic disorders in the offspring, whereas paternal stress can affect offspring behavioral development [92,93,94,95,96,97].

Despite the primary role of epididymosomes in the modeling of the spermatozoa ncRNA payload during the epididymal journey, few notions have been reported regarding circRNA cargo modulation. Sun and co-authors performed RNA-seq experiments to profile circRNAs in adult donkey testis compared with the *caput* epididymis. They identified totals of 12.648 and 6.261 circRNAs in the testis and *caput* epididymides, respectively. Among them, 3.928 circRNAs are shared by the two tissues. By contrast, 1.971 circRNAs are differentially expressed in the testis, with 499 up- and 1.472 downregulated circRNAs compared with the *caput* epididymis [53]. Functionally, bioinformatic analyses revealed the involvement of DE-circRNAs in GnRH, estrogen and calcium signaling pathways [53]. Li et al. investigated the epididymal circRNA landscape between yaks and cattle yaks. They identified 1.298 circRNAs shared in both types of epididymis, of which 137 were differentially expressed between the two species and potentially involved in the regulation of reproductive functions and spermatozoa activities [98]. Paternal exposure to HFD induces 109 DE-circRNAs in HFD spermatozoa, consisting of 43 up- and 66 downregulated [14]. Interestingly, while upregulated circRNAs appear molecularly dependent on the enhanced ability of HFD spermatozoa to perform endogenous backsplicing, the downregulated counterpart is dependent on an inefficient epididymal circRNA biogenesis and delivery. In mice, the FUS protein, localized for the first time in the epididymal Principal cells, is physically and functionally connected to the QKI protein to modulate backsplicing [14]. In the HFD condition, the loss of the typical multilayered columnar arrangement of the epididymal Principal cells negatively affects the epididymal epithelial architecture needed to coordinate protein-to-protein interactions [10,19,32,99] and the proper FUS–QKI physical interaction underling epididymal circRNA biogenesis [14]. In addition, in vitro experiments that promoted the transfer of epididymosome-derived circSMAD2 into sperm cells induced the recovery of circSMAD2-dependent ceRNET, which positively modulated sperm motility, and thus, experimentally confirmed the key role of epididymosome-mediated circRNAs in the field of male fertility.

**Figure 2:**
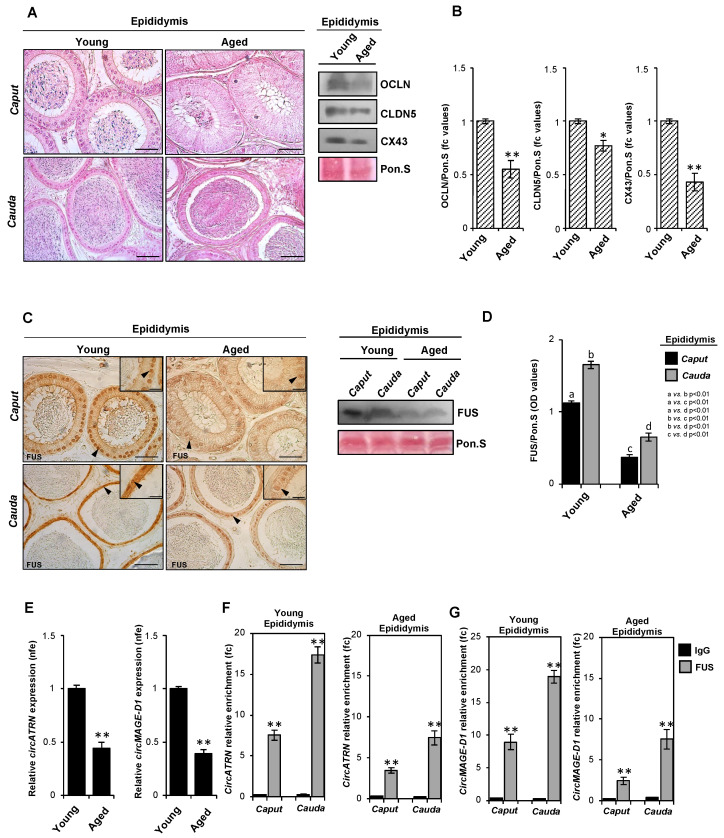
Characterization of backsplicing machinery in Aged epididymis. (**A**) H&E staining of Bouin’s fixed *caput* and *cauda* epididymides (sections: 7 μm; scale bar: 50 μm) collected from Young and Aged mice (sample size: 5 different samples for each experimental group). (**B**) Western blot analysis of OCLN, CLDN5 and CX43 proteins in Young and Aged epididymides (sample size: 5 different samples for each experimental group). Signals were quantified by densitometry analysis and normalized to Ponceau Red (Pon.S). Data are expressed in fc values and reported as mean ± SEM. **: *p* < 0.01; *: *p* < 0.05. (**C**) Immunocytochemistry of FUS in Bouin’s fixed *caput* and *cauda* epididymis of Young and Aged mice (sections: 7 μm) (sample size: 5 different samples for each experimental group). The FUS protein localization in Principal cells is indicated by black arrows (scale bar: 50 μm; inset scale: 20 μm). (**D**) Western blot analysis of FUS protein in *caput* and *cauda* epididymides of Young and Aged mice (sample size: 5 different samples for each experimental group). Signals were quantified by densitometry analysis and normalized to Ponceau Red (Pon.S). Data are expressed in OD values and reported as the mean ± SEM. Experimental groups with statistically significant differences (*p* < 0.01) were indicated with different letters. (**E**) Expression analysis of circATRN and circMAGE-D1 in Young and Aged epididymides (sample size: 5 different samples for each experimental group). qRT-PCR data were normalized using cyclophilin, expressed as fold expression (nfe) and reported as mean value ± S.E.M. **: *p* < 0.01. (**F**,**G**) The enrichment levels of circATRN and circMAGE-D1 in RIP assay (FUS-IP compared with IgG-IP) performed in *caput* and *cauda* epididymides of Young and Aged mice. qRT-PCR data are reported as the mean ± SEM from three independent experiments. ** *p* < 0.01. For all investigations, the Shapiro–Wilk test was used to assess the data normality and to confirm the normal distribution of data. Following the data confirmation, Student’s *t*-test (for two independent group comparisons) was used to identify the groups with different means. Differences with *p* < 0.05 were considered statistically significant (Manfrevola, F; Chioccarelli, T; Cobellis, G; Chianese, R. (University of Campania “Luigi Vanvitelli”, 80138 Naples, Italy). Unpublished work, 2025 [101]).

In the current review, taking advantage of the experimental mouse model of aging, we provide new unpublished findings on the (i) epididymal backsplicing mechanism and (ii) epididymal expression of molecular actors that promote backsplicing. This experimental model represents an excellent physio-pathological condition with affected male fertility. In fact, it has recently been reported that the mouse model of aging shows (i) decreased spermatogonia proliferation, (ii) testicular germ cell depletion, (iii) decreased sperm number, (iv) decreased sperm motility, (v) increased morphological sperm anomalies, (vi) somatic cell senescence in both the testis and epididymis, and (vii) epididymal epithelial layers defects [95,100]. In order to add a piece of knowledge to what has already been demonstrated, we first evaluated the morphology of the epididymis collected from Young vs. Aged mice. As shown by hematoxylin and eosin (H&E) staining (Figure 2A), an anomalous epididymal morphology characterized by a severe disorganization in the layered epithelium, which mainly occurred in the *caput* epididymal segment, was observed in the Aged epididymis. To assess the integrity of the BEB, the expression analysis of epithelial junction markers was carried out in the Young vs. Aged epididymis by Western blot analysis. The results show a significant reduction in all the epithelial junction markers analyzed (Figure 2B). Regarding tight junctions, we observed lower expression levels of OCLN and CLDN5 proteins in the Aged than the Young epididymis. Similarly, a significant reduction in CX43 protein, which is a marker of epithelial gap junctions, occurred in the Aged epididymis in comparison with the Young one, demonstrating a complete deregulation of BEB epithelial junctions (Manfrevola, F; Chioccarelli, T; Cobellis, G; Chianese, R. (University of Campania “Luigi Vanvitelli”, 80138 Naples, Italy). Unpublished work, 2025 [101]). To investigate whether the damaged epididymal epithelial architecture could compromise epididymal backsplicing, we morphologically characterized the FUS protein in the epididymal epithelium. Immunocytochemistry analysis performed in *caput* and *cauda* epididymal sections of Young vs. Aged mice using the FUS antibody showed a well-defined FUS localization in epididymal Principal cells that progressively increased from the *caput* to the *cauda* in the Young epididymis (Figure 2C). Although the same trend occurred in the Aged epididymis, an evident reduction in the intensity of the FUS signal was observed, suggesting that the aging promoted the reduction in the epididymal FUS content likely affected the biogenesis of FUS-dependent epididymal-derived circRNAs. Accordingly, a significant increase in the FUS protein from the *caput* to *cauda* sections, though less pronounced in the Aged epididymis, was demonstrated by Western blot analysis (Figure 2D) (Manfrevola, F; Chioccarelli, T; Cobellis, G; Chianese, R. (University of Campania “Luigi Vanvitelli”, 80138 Naples, Italy). Unpublished work, 2025 [101]). 

In order to assess the potential defective epididymal backsplicing, we analyzed the expression levels of circATRN and circMAGE-D1, which have been reported to be downregulated in the epididymis of the HFD model [14]. The expression of both circRNAs was significantly reduced in the Aged compared with the Young epididymis (Figure 2E), suggesting that the aging compromised the epididymal circRNA biogenesis. To confirm this hypothesis, we carried out an RNA-Binding Protein Immunoprecipitation (RIP) Assay in the Aged epididymis by using the FUS antibody. Once again, circATRN and circMAGE-D1 were chosen as representative circRNAs downregulated in the Aged epididymis. Relative to the use of the IgG control, significant 7.56- and 17.34-fold enrichments of circATRN were observed when the anti-FUS antibody was used in the *caput* and *cauda* Young epididymides, respectively (Figure 2F). Despite a similar trend, which consisted of significant 3.45- and 7.45-fold enrichments of circATRN, observed in the *caput* and *cauda* Aged epididymis, respectively, a less efficient backsplicing was highlighted (Figure 2F). The RIP experiments for circMAGE-D1 recapitulated this molecular trend. Indeed, significant 8.94- and 18.94-fold enrichments of circMAGE-D1 occurred in the Young *caput* and *cauda* epididymides, respectively, whereas a less efficient enrichment of circMAGE-D1 (2.46-fold in the *caput* and 7.54-fold in the *cauda*) was observed in the Aged epididymis (Figure 2G) (Manfrevola, F; Chioccarelli, T; Cobellis, G; Chianese, R. (University of Campania “Luigi Vanvitelli”, 80138 Naples, Italy). Unpublished work, 2025 [101]). 

Collectively, these preliminary data provide compelling evidence for the epididymal backsplicing mechanism, highlighting the tight association between the correct tissue morphology and its molecular functionality.

## 6. Conclusions

**Figure 3:**
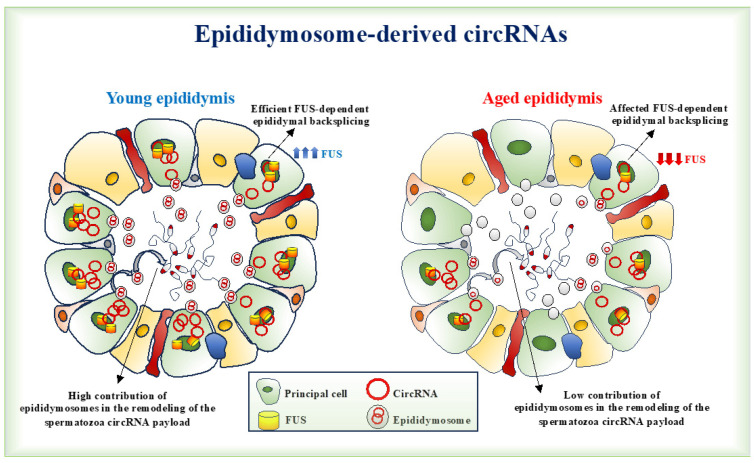
Aging affects epididymal backsplicing: a schematic view. In Young epididymis, FUS-dependent epididymal backsplicing promotes the efficient remodeling of the spermatozoa circRNA payload via epididymosomes delivery (**left side**). In Aged epididymis, the reduction of FUS protein in Principal cells impairs epididymal backsplicing and, in turn, the delivery of epididymosome-derived circRNAs to the spermatozoa (**right side**).

The epididymis drives sperm maturation, allowing for the acquisition of new functional skills and changing the sperm molecular and epigenetic payload. In the context of sperm RNA cargo, circRNAs have gained considerable attention in recent years. Here, we aimed to shed light on the epididymal backsplicing and, in turn, on the role of epididymosomes in the remodeling of the sperm circRNA payload. Interestingly, bringing attention to aging, we characterized FUS-dependent epididymal backsplicing, which suggested impaired epididymal circRNA biogenesis in a mouse model of aging (Figure 3).

## Figures and Tables

**Table 1:** CircRNA implications in male fertility.

Biological Target	Functional Implications	References
Germ cells	*CircRNAs implicated in control of male germ cell lineage*	[60]
Spermatogenesis	*CircRNAs implicated in spermatogenesis and testis development*	[61,62,63,64]
Sperm morpho-functional skills	*CircRNAs implicated in sperm morphology and motility*	[11,65,66]
Zygote	*CircRNAs implicated in embryo development*	[67,68,69,70,71]

**Table 2:** Pathological conditions that affect circRNAs involved in male fertility regulation.

Pathological Condition	Functional Implications	References
Asthenozoospermia	*CircRNAs implicated in sperm motility pathways*	[12,13,65,66,77]
Non-obstructive azoospermia (NOA)	*CircRNAs implicated in axoneme assembly and microtubule-based pathways*	[55,78,79,80]
Sertoli cell-only syndrome (SCOS)	*CircRNAs implicated in Sertoli cell and microenvironmental dysfunctions*	[52]
Obesity	*CircRNAs implicated in sperm oxidative stress pathways*	[14,15]
Advanced paternal age (APA)	*CircRNAs implicated in DNA repair and meiotic recombination pathways*	[81]

## Data Availability

The raw data this study are available from the corresponding author upon reasonable request.

## Abbreviations

The following abbreviations are used in this manuscript:

Non-coding RNAs	ncRNAs
Circular RNAs	circRNAs
Differentially expressed circRNAs	DE-circRNAs
BEB	Blood–Epididymal Barrier
Tight junctions	TJs
Claudin	CLDN
Zona occludens	ZO
Occludin	OCLN
Connexin43	CX43
RNA-binding proteins	RBPs
Fused in sarcoma	FUS
Quaking	QKI
RNA polymerase II	RNApol2
CircRNA/miRNA/mRNA network	ceRNET
Non-obstructive azoospermia	NOA
Sertoli cell-only syndrome	SCOS
High-fat diet	HFD
Advanced paternal age	APA
Hematoxylin and eosin	H&E
RNA-Binding Protein Immunoprecipitation	RIP
Quantitative RT-PCR	RT-qPCR

## References

[1] Binato de Souza A.P., Schorr-Lenz A.M., Lucca F., Bustamante-Filho I.C. (2017). The epididymis and its role on sperm quality and male fertility. Anim. Reprod..

[2] Elbashir S., Magdi Y., Rashed A., Henkel R., Agarwal A. (2021). Epididymal contribution to male infertility: An overlooked problem. Andrologia.

[3] Gervasi M.G., Visconti P.E. (2017). Molecular changes and signaling events occurring in spermatozoa during epididymal maturation. Andrology.

[4] James E.R., Carrell D.T., Aston K.I., Jenkins T.G., Yeste M., Salas-Huetos A. (2020). The Role of the Epididymis and the Contribution of Epididymosomes to Mammalian Reproduction. Int. J. Mol. Sci..

[5] Trigg N.A., Eamens A.L., Nixon B. (2019). The contribution of epididymosomes to the sperm small RNA profile. Reproduction.

[6] Sullivan R., Saez F. (2013). Epididymosomes, prostasomes, and liposomes: Their roles in mammalian male reproductive physiology. Reproduction.

[7] Sullivan R. (2015). Epididymosomes: A heterogeneous population of microvesicles with multiple functions in sperm maturation and storage. Asian J. Androl..

[8] Nixon B., De Iuliis G.N., Dun M.D., Zhou W., Trigg N.A., Eamens A.L. (2019). Profiling of epididymal small non-protein-coding RNAs. Andrology.

[9] Méar L.O., Tsai P.S., Tamessar C.T., Schjenken J.S., Nixon B. (2024). Epididymosomes: Composition and Functions for Sperm Maturation. Adv. Anat. Embryol. Cell Biol..

[10] Zhou W., De Iuliis G.N., Dun M.D., Nixon B. (2018). Characteristics of the epididymal luminal environment responsible for sperm maturation and storage. Front. Endocrinol..

[11] Chioccarelli T., Manfrevola F., Ferraro B., Sellitto C., Cobellis G., Migliaccio M., Fasano S., Pierantoni R., Chianese R. (2019). Expression Patterns of CircularRNAs in High Quality and Poor Quality Human Spermatozoa. Front. Endocrinol..

[12] Manfrevola F., Chioccarelli T., Cobellis G., Fasano S., Ferraro B., Sellitto C., Marella G., Pierantoni R., Chianese R. (2020). CircRNA Role and circRNA-Dependent Network (ceRNET) in Asthenozoospermia. Front. Endocrinol..

[13] Manfrevola F., Ferraro B., Sellitto C., Rocco D., Fasano S., Pierantoni R., Chianese R. (2021). CRISP2, CATSPER1 and PATE1 Expression in Human Asthenozoospermic Semen. Cells.

[14] Manfrevola F., Chioccarelli T., Mele V.G., Porreca V., Mattia M., Cimini D., D’Agostino A., Cobellis G., Fasano S., Schiraldi C. (2023). Novel Insights into circRNA Saga Coming from Spermatozoa and Epididymis of HFD Mice. Int. J. Mol. Sci..

[15] Mele V.G., Chioccarelli T., Finamore R., D’Agostino A., D’Agostino M., Cimini D., Mattia M., Porreca V., Giori A.M., Fasano S. (2023). Antioxidants positively regulate obesity dependent circRNAs-sperm quality-functional axis. Front. Endocrinol..

[16] Manfrevola F., Potenza N., Chioccarelli T., Di Palo A., Siniscalchi C., Porreca V., Scialla A., Mele V.G., Petito G., Russo A. (2022). Actin remodeling driven by circLIMA1: Sperm cell as an intriguing cellular model. Int. J. Biol. Sci..

[17] Mele V.G., Chioccarelli T., Diano N., Cappetta D., Ferraro B., Telesca M., Moggio M., Porreca V., De Angelis A., Berrino L. (2024). Variation of sperm quality and circular RNA content in men exposed to environmental contamination with heavy metals in ’Land of Fires’, Italy. Hum. Reprod..

[18] Jones R., Lopez K.H. (2004). Human Reproductive Biology.

[19] Cornwall G.A. (2009). New insights into epididymal biology and function. Hum. Reprod. Updat..

[20] Murashima A., Bingfang X., Hinton B.T. (2015). Understanding normal and abnormal development of the Wolffian/epididymal duct by using transgenic mice. Asian J. Androl..

[21] De Mello Santos T., Hinton B.T. (2019). We, the developing rete testis, efferent ducts, and Wolffian duct, all hereby agree that we need to connect. Andrology.

[22] Breton S., Nair A.V., Battistone M.A. (2019). Epithelial dynamics in the epididymis: Role in the maturation, protection, and storage of Spermatozoa. Andrology.

[23] Adamali H.I., Hermo L. (1996). Apical and narrow cells are distinct cell types differing in their structure, distribution, and functions in the adult rat epididymis. J. Androl..

[24] Seiler P., Wenzel I., Wagenfeld A., Yeung C.H., Nieschlag E., Cooper T.G. (1998). The appearance of basal cells in the developing murine epididymis and their temporal expression of macrophage antigens. Int. J. Androl..

[25] Mandon M., Hermo L., Cyr D.G. (2015). Isolated Rat Epididymal Basal Cells Share Common Properties with Adult Stem Cells. Biol. Reprod..

[26] Leung G.P., Cheung K.H., Leung C.T., Tsang M.W., Wong P.Y. (2004). Regulation of epididymal principal cell functions by basal cells: Role of transient receptor potential (Trp) proteins and cyclooxygenase-1 (COX-1). Mol. Cell Endocrinol..

[27] Robaire B., Hinton B.T., Orgebin-Crist M., Neill K. (2006). The Epididymis. Knobil and Neill’s Physiology of Reproduction.

[28] Shum W.W., Ruan Y.C., Da Silva N., Breton S. (2011). Establishment of cell-cell cross talk in the epididymis: Control of luminal acidification. J. Androl..

[29] Dufresne J., Gregory M., Pinel L., Cyr D.G. (2024). Three-Dimensional Cell Culture of Epididymal Basal Cells and Organoids: A Novel Tool for Toxicology. Curr. Protoc..

[30] Park Y.J., Battistone M.A., Kim B., Breton S. (2017). Relative contribution of clear cells and principal cells to luminal pH in the mouse epididymis. Biol. Reprod..

[31] Mital P., Hinton B.T., Dufour J.M. (2011). The Blood-Testis and Blood-Epididymis Barriers Are More than Just Their Tight Junctions. Biol. Reprod..

[32] Manfrevola F., Martinez G., Coutton C., Rocco D., Reynaud K., Le Vern Y., Froment P., Beauclair L., Aubert D., Pierantoni R. (2021). Ankrd31 in Sperm and Epididymal Integrity. Front. Cell. Dev. Biol..

[33] Dubé E., Dufresne J., Chan P.T.K., Hermo L., Cyr D.G. (2010). Assessing the role of claudins in maintaining the integrity of epididymal tight junctions using novel human epididymal cell lines. Biol. Reprod..

[34] Cyr D.G., Hermo L., Blaschuk O.W., Robaire B. (1992). Distribution and regulation of epithelial-cadherin messenger ribonucleic acid and immunocytochemical localization of epithelial cadherin in the rat epididymis. Endocrinology.

[35] Cyr D.G., Gregory M., Dubé E., Dufresne J., Chan P.T.K., Hermo L. (2007). Orchestration of occludins, claudins, catenins and cadherins as players involved in maintenance of the blood-epididymal barrier in animals and humans. Asian J. Androl..

[36] Cyr D.G. (2011). Connexins and pannexins. coordinating cellular communication in the testis and epididymis. Spermatogenesis.

[37] Elfgen V., Mietens A., Mewe M., Hau T., Middendorff R. (2018). Contractility of the epididymal duct: Function, regulation and potential drug effects. Reproduction.

[38] Töpfer-Petersen E., Petrounkina A.M., Ekhlasi-Hundrieser M. (2000). Oocyte-sperm interactions. Anim. Reprod. Sci..

[39] Maldera J.A., Weigel Muñoz M., Chirinos M., Busso D., Raffo F.G.E., Battistone M.A., Blaquier J.A., Larrea F., Cuasnicu P.S. (2014). Human fertilization: Epididymal hCRISP1 mediates sperm-zona pellucida binding through its interaction with ZP3. Mol. Hum. Reprod..

[40] Skerget S., Rosenow M.A., Petritis K., Karr T.L. (2015). Sperm Proteome Maturation in the Mouse Epididymis. PLoS ONE.

[41] Wong G.E., Zhu X., Prater C.E., Oh E., Evans J.P. (2001). Analysis of fertilin alpha (ADAM1)-mediated sperm-egg cell adhesion during fertilization and identification of an adhesion-mediating sequence in the disintegrin-like domain. J. Biol. Chem..

[42] Sullivan R., Mieusset R. (2016). The human epididymis: Its function in sperm maturation. Hum. Reprod. Updat..

[43] Martinez G., Cappetta D., Telesca M., Urbanek K., Castaldo G., Dhellemmes M., Mele V.G., Chioccarelli T., Porreca V., Barbotin A.L. (2023). Cytochalasin D restores nuclear size acting on F-actin and IZUMO1 localization in low-quality spermatozoa. Int. J. Biol. Sci..

[44] O’Flaherty C. (2019). Orchestrating the antioxidant defenses in the epididymis. Andrology.

[45] Chianese R., Pierantoni R. (2021). Mitochondrial Reactive Oxygen Species (ROS) Production Alters Sperm Quality. Antioxidants.

[46] Salzman J., Gawad C., Wang P.L., Lacayo N., Brown P.O. (2012). Circular RNAs Are the Predominant Transcript Isoform from Hundreds of Human Genes in Diverse Cell Types. PLoS ONE.

[47] Chen L.L., Yang L. (2015). Regulation of circRNA biogenesis. RNA Biol..

[48] Li X., Yang L., Chen L.L. (2018). The Biogenesis, Functions, and Challenges of Circular RNAs. Mol. Cell..

[49] Dong W.W., Li H.M., Qing X.R., Huang D.H., Li H.G. (2016). Identification and characterization of human testis derived circular RNAs and their existence in seminal plasma. Sci. Rep..

[50] Lin X., Han M., Cheng L., Chen J., Zhang Z., Shen T., Wang M., Wen B., Ni T., Han C. (2016). Expression dynamics, relationships, and transcriptional regulations of diverse transcripts in mouse spermatogenic cells. RNA Biol..

[51] Zhou T., Xie X., Li M., Shi J., Zhou J.J., Knox K.S., Wang T., Chen Q., Gu W. (2018). Rat BodyMap transcriptomes reveal unique circular RNA features across tissue types and developmental stages. RNA.

[52] Zhu F., Luo Y., Bo H., Gong G., Tang R., Fan J., Zhang H., Liu G., Zhu W., Tan Y. (2021). Trace the profile and function of circular RNAs in Sertoli cell only syndrome. Genomics.

[53] Sun Y., Wang Y., Li Y., Akhtar F., Wang C., Zhang Q. (2022). Identification of Circular RNAs of Testis and Caput Epididymis and Prediction of Their Potential Functional Roles in Donkeys. Genes.

[54] Yue D., Yang R., Xiong C., Yang R. (2022). Functional prediction and profiling of exosomal circRNAs derived from seminal plasma for the diagnosis and treatment of oligoasthenospermia. Exp. Ther. Med..

[55] Zhang Z., Wu H., Zheng L., Zhang H., Yang Y., Mao J., Liu D., Zhao L., Liang H., Jiang H. (2022). Identification and characterization of circular RNAs in the testicular tissue of patients with non-obstructive azoospermia. Asian J. Androl..

[56] La Y., Ma X., Bao P., Chu M., Yan P., Liang C., Guo X. (2023). Genome-wide landscape of mRNAs, lncRNAs, and circRNAs during testicular development of yak. Int. J. Mol. Sci..

[57] Babakhanzadeh E., Hoseininasab F.A., Khodadadian A., Nazari M., Hajati R., Ghafouri-Fard S. (2024). Circular RNAs: Novel noncoding players in male infertility. Hereditas.

[58] Yan Q., Wang Q. (2025). Exploring the Characters of Non-Coding RNAs in Spermatogenesis and Male Infertility. Int. J. Mol. Sci..

[59] Saberiyan M., Karimi E., Safi A., Movahhed P., Dehdehi L., Haririan N., Mirfakhraie R. (2023). Circular RNAs: Novel Biomarkers in Spermatogenesis Defects and Male Infertility. Reprod. Sci..

[60] Zhou F., Chen W., Jiang Y., He Z. (2019). Regulation of long non-coding RNAs and circular RNAs in spermatogonial stem cells. Reproduction.

[61] Tang W., Xu Q.H., Chen X., Guo W., Ao Z., Fu K., Ji T., Zou Y., Chen J.J., Zhang Y. (2023). Transcriptome sequencing reveals the effects of circRNA on testicular development and spermatogenesis in Qianbei Ma Goats. Front. Vet. Sci..

[62] Sahlu B.W., Wang H., Hu Z., Heng N., Gong J., Wang H., Zhu H., Zhao S. (2023). Identification of a circRNA-miRNA-mRNA network to explore the effects of circRNAs on Holstein bull testis after sexual maturity. Anim. Reprod. Sci..

[63] Zhang F., Zhang X., Ning W., Zhang X., Ru Z., Wang S., Sheng M., Zhang J., Zhang X., Luo H. (2021). Expression analysis of circular RNAs in Young and sexually mature boar testes. Animals.

[64] Sahoo B., Gupta M.K. (2024). Transcriptome Analysis Reveals Spermatogenesis-Related CircRNAs and LncRNAs in Goat Spermatozoa. Biochem. Genet..

[65] El-Gamal R., Zalata A., Mazroa S.A., Comhaire F., Gamal A., Shaker O.G., Hazem N.M. (2024). Evaluation of circANKLE2 & circL3MBTL4-RNAs Expression in Fertile and Infertile Men. Biochem. Genet..

[66] Cheng L., Jin H., Xiao T., Yang X., Zhao T., Xu E.Y. (2024). Human circBOULE RNAs as potential biomarkers for sperm quality and male infertility. J. Biomed. Res..

[67] Ragusa M., Barbagallo D., Chioccarelli T., Manfrevola F., Cobellis G., Di Pietro C., Brex D., Battaglia R., Fasano S., Ferraro B. (2019). CircNAPEPLD is expressed in human and murine spermatozoa and physically interacts with oocyte miRNAs. RNA Biol..

[68] Fan X., Zhang X., Wu X., Guo H., Hu Y., Tang F., Huang Y. (2015). Single-cell RNA-seq transcriptome analysis of linear and circular RNAs in mouse preimplantation embryos. Genome Biol..

[69] Dang Y., Yan L., Hu B., Fan X., Ren Y., Li R., Lian Y., Yan J., Li Q., Zhang Y. (2016). Tracing the expression of circular RNAs in human pre-implantation embryos. Genome Biol..

[70] Zhang S., Ding Y., He J., Zhang J., Liu X., Chen X., Su Y., Wang Y., Gao R. (2018). Altered expression patterns of circular RNAs between implantation sites and interimplantation sites in early pregnant mice. J. Cell Physiol..

[71] Chioccarelli T., Falco G., Cappetta D., De Angelis A., Roberto L., Addeo M., Ragusa M., Barbagallo D., Berrino L., Purrello M. (2021). FUS driven circCNOT6L biogenesis in mouse and human spermatozoa supports zygote development. Cell Mol. Life Sci..

[72] Loux S.C., Crawford K.R., Ing N.H., González-Fernández L., Macías-García B., Love C.C., Varner D.D., Velez I.C., Choi Y.H., Hinrichs K. (2013). CatSper and the relationship of hyperactivated motility to intracellular calcium and pH kinetics in equine sperm. Biol. Reprod..

[73] Avenarius M.R., Hildebrand M.S., Zhang Y., Meyer N.C., Smith L.L., Kahrizi K., Najmabadi H., Smith R.J. (2009). Human male infertility caused by mutations in the CATSPER1 channel protein. Am. J. Hum. Genet..

[74] Liu F.J., Liu X., Han J.L., Wang Y.W., Jin S.H., Liu X.X., Liu J., Wang W.T., Wang W.J. (2015). Aged men share the sperm protein PATE1 defect with young asthenozoospermia patients. Hum. Reprod..

[75] Zhang S., Wang Q.M., Ding X.P., Wang T., Mu X.M., Chen Z.Y. (2016). Association of polymorphisms in PATE1 gene with idiopathic asthenozoospermia in Sichuan, China. J. Reprod. Immunol..

[76] Heidary Z., Zaki-Dizaji M., Saliminejad K., Khorramkhorshid H.R. (2019). Expression analysis of the CRISP2, CATSPER1, PATE1 and SEMG1 in the sperm of men with idiopathic asthenozoospermia. J. Reprod. Infertility..

[77] Wu Y., Li H., Zhao X., Baki G., Ma C., Yao Y., Li J., Yao Y., Wang L. (2022). Differential expression of circRNAs of testes with high and low sperm motility in Yili geese. Front. Genet..

[78] Ge P., Zhang J., Zhou L., Lv M.Q., Li Y.X., Wang J., Zhou D.X. (2019). CircRNA expression profile and functional analysis in testicular tissue of patients with non-obstructive azoospermia. Reprod. Biol. Endocrinol..

[79] Bo H., Liu Z., Tang R., Gong G., Wang X., Zhang H., Zhu F., Zhou D., Zhu W., Tan Y. (2020). Testicular biopsies microarray analysis reveals circRNAs are involved in the pathogenesis of non-obstructive azoospermia. Aging.

[80] Liu L., Li F., Wen Z., Li T., Lv M., Zhao X., Zhang W., Liu J., Wang L., Ma X. (2020). Preliminary investigation of the function of hsa_circ_0049356 in nonobstructive azoospermia patients. Andrologia.

[81] Zhou Q., Liu A., Ji H., Ji J., Sun J., Ling Z., Li G., Ling X., Xu L., Chen X. (2023). Expression profiles of circular RNAs in spermatozoa from aging men. Mol. Biol. Rep..

[82] Zhou W., Stanger S.J., Anderson A.L., Bernstein I.R., De Iuliis G.N., McCluskey A., McLaughlin E.A., Dun M.D., Nixon B. (2019). Mechanisms of tethering and cargo transfer during epididymosome-sperm interactions. BMC Biol..

[83] Reilly J.N., McLaughlin E.A., Stanger S.J., Anderson A.L., Hutcheon K., Church K., Mihalas B.P., Tyagi S., Holt J.E., Eamens A.L. (2016). Characterisation of mouse epididymosomes reveals a complex profile of microRNAs and a potential mechanism for modification of the sperm epigenome. Sci. Rep..

[84] Sharma U., Conine C.C., Shea J.M., Boskovic A., Derr A.G., Bing X.Y., Belleannee C., Kucukural A., Serra R.W., Sun F. (2016). Biogenesis and function of tRNA fragments during sperm maturation and fertilization in mammals. Science.

[85] Sharma U., Sun F., Conine C.C., Reichholf B., Kukreja S., Herzog V.A., Ameres S.L., Rando O.J. (2018). Small RNAs Are Trafficked from the Epididymis to Developing Mammalian Sperm. Dev. Cell..

[86] Conine C.C., Sun F., Song L., Rivera-Pérez J.A., Rando O.J. (2018). Small RNAs Gained during Epididymal Transit of Sperm Are Essential for Embryonic Development in Mice. Dev. Cell..

[87] Chen Q., Yan M., Cao Z., Li X., Zhang Y., Shi J., Feng G.H., Peng H., Zhang X., Zhang Y. (2016). Sperm tsRNAs contribute to intergenerational inheritance of an acquired metabolic disorder. Science.

[88] Boskovic A., Bing X.Y., Kaymak E., Rando O.J. (2020). Control of noncoding RNA production and histone levels by a 5′ tRNA fragment. Genes Dev..

[89] Zhang S., Yu M., Liu C., Wang L., Hu Y., Bai Y., Hua J. (2012). MIR-34c regulates mouse embryonic stem cells differentiation into male germ-like cells through RARg. Cell. Biochem. Funct..

[90] Yuan S., Tang C., Zhang Y., Wu J., Bao J., Zheng H., Xu C., Yan W. (2015). mir-34b/c and mir-449a/b/c are required for spermatogenesis, but not for the first cleavage division in mice. Biol. Open.

[91] Wang B., Wang Y., Zhang M., Du Y., Zhang Y., Xing X., Zhang L., Su J., Zhang Y., Zheng Y. (2014). MicroRNA-34c expression in donor cells influences the early development of somatic cell nuclear transfer bovine embryos. Cell Reprogram..

[92] Zhang Y., Zhang X., Shi J., Tuorto F., Li X., Liu Y., Liebers R., Zhang L., Qu Y., Qian J. (2018). Dnmt2 mediates intergenerational transmission of paternally acquired metabolic disorders through sperm small non-coding RNAs. Nat. Cell Biol..

[93] Short A.K., Yeshurun S., Powell R., Perreau V.M., Fox A., Kim J.H., Pang T.Y., Hannan A.J. (2017). Exercise alters mouse sperm small noncoding RNAs and induces a transgenerational modification of male offspring conditioned fear and anxiety. Transl. Psychiatry.

[94] Grandjean V., Fourré S., De Abreu D.A., Derieppe M.A., Remy J.J., Rassoulzadegan M. (2015). RNA-mediated paternal heredity of diet-induced obesity and metabolic disorders. Sci. Rep..

[95] Dickson D.A., Paulus J.K., Mensah V., Lem J., Saavedra-Rodriguez L., Gentry A., Pagidas K., Feig L.A. (2018). Reduced levels of miRNAs 449 and 34 in sperm of mice and men exposed to early life stress. Transl. Psychiatry.

[96] Chan J.C., Morgan C.P., Adrian Leu N., Shetty A., Cisse Y.M., Nugent B.M., Morrison K.E., Jašarević E., Huang W., Kanyuch N. (2020). Reproductive tract extracellular vesicles are sufficient to transmit intergenerational stress and program neurodevelopment. Nat. Commun..

[97] de Castro Barbosa T., Ingerslev L.R., Alm P.S., Versteyhe S., Massart J., Rasmussen M., Donkin I., Sjögren R., Mudry J.M., Vetterli L. (2015). High-fat diet reprograms the epigenome of rat spermatozoa and transgenerationally affects metabolism of the offspring. Mol. Metab..

[98] Li C., Yan Y., Pan C., Adjei M., Shahzad K., Wang P., Pan M., Li K., Wang Y., Zhao W. (2023). Identification and analysis of differentially expressed (DE) circRNA in epididymis of yak and cattleyak. Front. Vet. Sci..

[99] Breton S., Ruan Y.C., Park Y.J., Kim B. (2016). Regulation of epithelial function, differentiation, and remodeling in the epididymis. Asian J. Androl..

[100] Endo T., Kobayashi K., Matsumura T., Emori C., Ozawa M., Kawamoto S., Okuzaki D., Shimada K., Miyata H., Shimada K. (2024). Multiple ageing effects on testicular/epididymal germ cells lead to decreased male fertility in mice. Commun. Biol..

[101] Manfrevola F., Chioccarelli T., Cobellis G., Chianese R.

